# Aggregation-induced fluorescent response of urea-bearing polyphenyleneethynylenes toward anion sensing

**DOI:** 10.1080/14686996.2021.1942982

**Published:** 2021-06-16

**Authors:** Jian Li, Muhammad Saleem, Qian Duan, Toyoji Kakuchi, Yougen Chen

**Affiliations:** a Institute for Advanced Study, Shenzhen University, Shenzhen, China; b College of Physics and Optoelectronic Engineering, Shenzhen University, Shenzhen, China; c School of Materials Science and Engineering, Changchun University of Science and Technology, Jilin, China; d Frontier Chemistry Center, Faculty of Engineering, Hokkaido University, Sapporo, Japan

**Keywords:** Urea-bearing, Sonogashira coupling reaction, polyphenyleneethynylene, 20 Organic and soft materials (colloids, liquid crystals, gel, polymers)

## Abstract

A π-conjugated urea-bearing phenyleneethynylene polymer (**Poly-2**) was rationally designed by the Sonogashira coupling condensation reaction and had been demonstrated to have a unique fluorescent quenching effect for the optical detection of all determined anions, especially for CN^−^. The fluorescent emission of **Poly-2** was significantly quenched upon adding CN^−^, together accompanied with a continuous red shift of the emission peak from 442 to 464 nm with the cyanide concentration increased from 0 to 1.0 mM. On the contrary, its precursor polymer, **Poly-1**, itself also displayed fluorescent responsibility with all selected anions but had no obvious selectivity and tendency. For instance, the addition of highly basic CN^−^, N_3_^−^, AcO^−^, or F^−^ to **Poly-1** solution in DMF/H_2_O (v/v = 1:1) led to the photoluminescence amplification, while the addition of weakly basic anions like Cl^−^, I^−^, and Br^−^ showed a fluorescence quenching effect. Both polymers were in a seriously self-aggregated state in solution no matter in the absence or presence of an anion. Interestingly, it was found that **Poly-2** exhibited an aggregation-induced emission behavior, while **Poly-1** had an aggregation-caused quenching effect, based on the relationship between photoluminescence and polymer aggregation state. The structural characterizations were carried out by NMR spectroscopy and size exclusion chromatography measurements; the photoluminescence properties of **Poly-1** and **Poly-2** together with anion sensing properties were followed by fluorescence spectroscopy, and the relationship between photoluminescence and aggregation behavior of both polymers in solution was investigated by dynamic light scattering measurements.

## Graphical abstract

**Figure:**
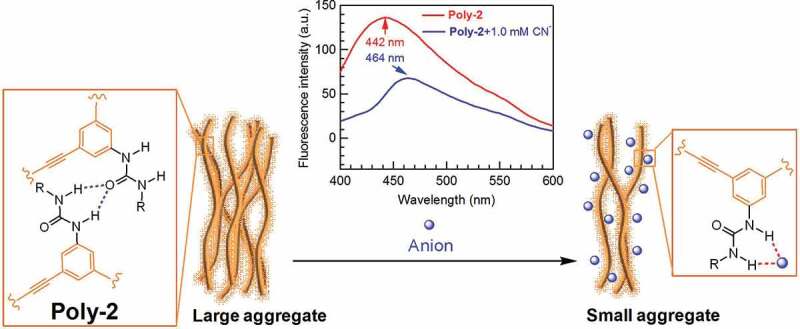

## Introduction

π-conjugated polymers exhibit useful optoelectric functions [1]. Over the past few decades, considerable efforts have been devoted to control the secondary and assembled structures of π-conjugated polymers, including poly(acetylene)s [2,3], poly(*p*-phenylene)s [4], poly(phenylenevinylene)s [5,6], poly(phenyleneethynylene)s [7,8], and poly(thiophene)s [9]. Poly(phenyleneethynylene)s and poly(thiophene)s substituted with optically active groups are representative polymers that form predominantly one-handed helices and/or chiral aggregates in the solution and solid states. Miyagi et al. have reported a series of optically active poly(phenyleneethynylene)s [10–12] and poly(thiophenyleneethynylene)s [13], and examined their chiral higher-order structures and optical properties. In the past, the helix sense control of polymers and aggregates has attracted much attention. Various attempts have been made to achieve chiral induction, amplification, memory, and switching in polymeric materials by utilizing chiral additives and initiators and by tuning temperature, solvent, pH, pressure, etc. Researchers are eager to amplify chirality in polymers because it is useful to induce a large chirality from a small amount of chiral source. The effects of chirality competition and cooperation at the side chains on higher-order structures will be a key step forward to achieve suitable π-conjugated monomers/polymers for advance applications.

Molecular sensing is a chemical analysis where a synthetic probe recognizes a given analyte producing a signal through the variation of defined and detectable properties [14]. Essential features of molecular sensing are the selectivity and efficiency of the chemical sensor [15]. Spectroscopic techniques based on chemosensors such as colorimetry and ﬂuorimetry have been especially appealing, and various concepts including the design of new chromoprobes or ﬂuoroprobes have been developed to improve sensitivity, selectivity, and the dynamic working range [16]. However, there is still a lot of improvement needed in terms of polymer structural design, such as specific ion binding. So, keeping in view the above-mentioned points, designing of new π-conjugated polymers still offers new opportunities and challenges.

Cyanide (HCN or CN^−^) is acutely toxic to mammals by all routes of administration, with a very steep and rate-dependent dose response curve that involves inactivation of cytochrome oxidase [17]. Strong interactions between CN^−^ and cytochrome C are known to interrupt the mitochondrial electron transfer cycle, leading to inhibition of oxidation metabolism, and cellular respiration processes. There are many international, national, and local regulations and guidelines regarding cyanide in the air, water and other media. The maximum contaminant level for cyanide set by the World Health Organization (WHO) in drinking water is 1.9 μM, and the maximum level in blood of human being was 23–26 μM [18]. There is a critical demand for a portable and rapid technique to diagnose cyanide exposure, because a large amount of cyanide estimated to be 1.5 million tons per year is widely used in industries such as gold mining, electroplating, and metallurgy and it can accumulate in foods and plants through inﬂow from polluted environments. So far, a copious number of studies have been reported for molecular sensing of cyanide, including electrochemical [19], colorimetric [20], fluorescent [21], and phosphorescent as-says [22]. In recent years, studies have reported fluorescent chemical sensors based on the strong nucleophilicity of CN^−^ exhibiting significant sensing performance and offering advantages such as high sensitivity and excellent selectivity with minimum analytic preparation [23,24].

**Scheme 1.**
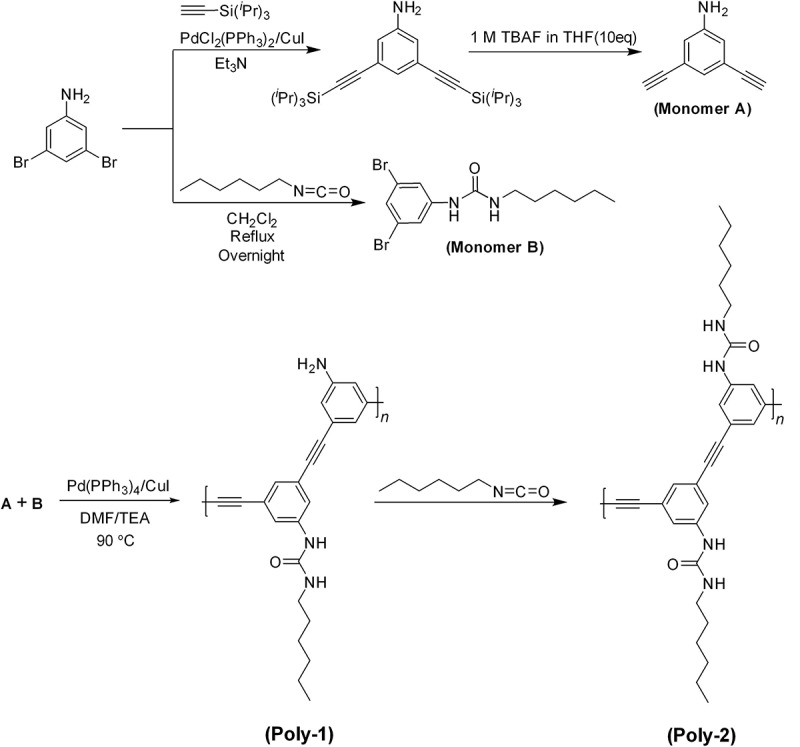
Synthesis of π-conjugated monomers A and B, and **Poly-1** and **Poly-2** by Sonogashira coupling reaction

Herein, we present synthesis of a new urea-bearing phenyleneethynylene polymer prepared by Sonogashira coupling method for analysis of several ions and specifically cyanide. The utilization of urea group to probe anions can be dated back to early 1990s, and a variety of urea-based anion receptors have been synthesized since Wilcox observed that the urea derivative could interact with phosphonates, sulfates and carboxylates in non-polar solvent [25]. Our approach combines specifically designed π-conjugated polymer with molecular sensing [26,27], where the florescence response is generated *via* Vander walls forces, π-electrons and hydrogen bonding (H-bond) interactions of anions as ligands and polymer as a specifically designed sensor. Florescence response resulting from a specific chemical interaction between the sensor, where the polymer with urea functional group as binding unit and alkyl chain as solubilizing entity, and different anions with specifically cyanide anion. As shown in Scheme 1, we designed the synthetic probes (**Poly-1** and **Poly-2**) as π-conjugated polymers based on a Sonogashira coupling method to exhibit selectivity florescence response towards CN^−^. **Poly-1** and **Poly-2** both showed anion sensing properties. In contrast, the target specificity of **Poly-2** specifically with CN^−^ shows very selective response. Our current investigation provides several key features, which not only limits to novel π-conjugated polymer synthesis but also polymer architecture offers specific florescence response for CN^−^. We believe our approach suggests a new polymer structural design and powerful analytic platform for diagnosis of cyanide in a real environment.

## Results and discussion

**Figure 1.**
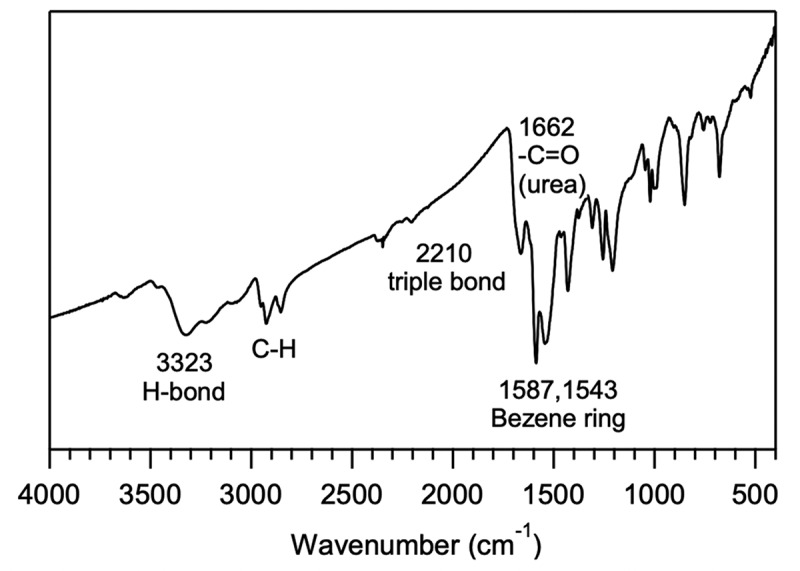
FT-IR spectrum of **Poly-2.**

For the polymer synthesis, the bifunctional monomers A and B were first synthesized from 3,5-dibromoaniline, in which monomer A was obtained by the Sonogashira reaction between 3,5-dibromoaniline and ethynyltriisopropylsilane followed by desilylation, and monomer B by directly reacting 3,5-dibromoaniline with *n*-hexyl isocyanate. The synthetic details and characterizations are described in Supporting Information (Figure S1-S3). The Sonogashira coupling reaction was first carried out between A and B to produce **Poly-1**, which was originally designed to have a high solubility in water or a mixed solvent with water for anion sensing. The polycondensation reaction produced **Poly-1** with a size exclusion chromatography (SEC) estimated number-average molecular weight (*M*_n,SEC_) of 13,500 g mol^−1^ and molecular weight distribution (*M*_w_/*M*_n_) of 2.42, whose SEC trace showed a broad monomodal distribution (Figure S4). The formation of **Poly-1** was also confirmed by the ^1^H NMR spectroscopy in DMSO-*d*_6_ with the broadening effect of proton signals, as shown in Figure S5a. The proton signals due to the urea, aromatic ring, amino, and hexyl groups were all observed correspondingly. It was found that **Poly-1** showed serious self-assembly property in solvent and was difficult to dissolve in pure water or DMF, but could have an acceptable solubility in DMF/water (v/v = 1:1). That is also why a highly dilute solution was used for SEC determination in DMF. Precisely for this reason, **Poly-1** was further reacted with *n*-hexyl isocyanate to chemically transform the naked amino group to urea group for decreasing the intermolecular hydrogen-bonding (H-bond) interactions and therefore enhancing the solubility of polymer. The complete chemical transformation of **Poly-1** to **Poly-2** was verified by the ^1^H NMR spectroscopy, as the signals due to amino group of **Poly-1** disappeared completely after the post modification. The *M*_n,SEC_ and *M*_w_/*M*_n_ of **Poly-2** were determined to be 6,080 g mol^−1^ and 1.43, respectively. Obviously, **Poly-2** with higher theoretical molecular weight had a lower hydrodynamic volume, as SEC measurements present in Figure S4. In addition, the shoulders attributed to lower molecular weight oligomers were also seen, due to its better solubility in DMF. The formation of H-bond was further evaluated by FT-IR using **Poly-2**, as shown in Figure 1. The characteristic absorption due to H-bond was clearly shown around 3323 cm^−1^. Moreover, the carbonyl group from urea group was obviously observed at 1662 cm^−1^, strongly indicating the formation of the desired **Poly-2**. Similarly, **Poly-1** should have experienced much stronger intermolecular aggregation due to the strong H-bond effect.

**Figure 2.**
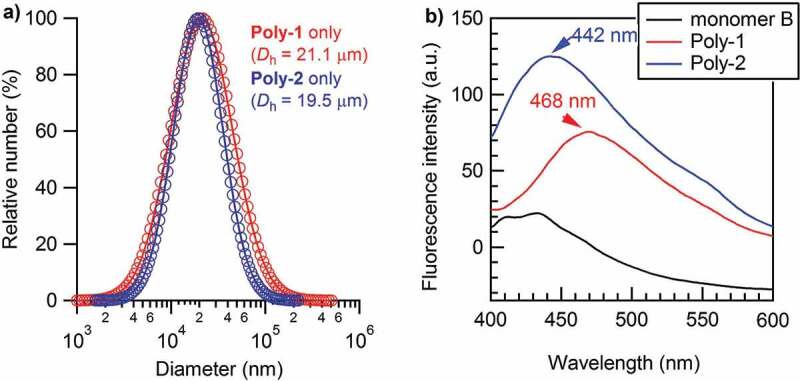
(a) Particle size distribution and average hydrodynamic diameter (*D*_h_) of **Poly-1** (red), and **Poly-2** (blue) determined by DLS in DMF/H_2_O (v/v = 1:1) mixed solvent at 25 °C under the weight concentration of 22 mg L^−1^ for both polymers (the concentration of urea group was 60 μM for **Poly-1** and 91 μM for **Poly-2**). (b) Fluorescence spectra of monomer B (black), **Poly-1** (blue), and **Poly-2** (red) in DMF/H_2_O (v/v = 1:1) at 25 °C and the excitation wavelength *λ*_ex_ = 350 nm under the same conditions with (a)

In order to elucidate the aggregation behavior of the two polymers, dynamic light scattering (DLS) measurements for both polymers were performed without addition of any anion at the weight concentration of 22 mg L^−1^ in DMF/H_2_O (v/v = 1:1), in which the concentration of urea group was 60 μM for **Poly-1** and 91 μM for **Poly-2** (Figure 2(a)). The solution for DLS determination was much more concentrated than that used for SEC determinations to obtain acceptable photoluminescence intensity for anion sensing, as discussed in the later section. It was rather clear that both polymers self-aggregated seriously at this concentration with their average hydrodynamic diameters (*D*_h_s) to be 21.1 and 19.5 μm, respectively, and the *D*_h_ distribution ranging from several micrometers to hundreds of micrometers. **Poly-1** showed a broader size distribution and more serious aggregation due to the sterically unhindered amino group providing more favorable H-bond interactions, which should also be the reason that **Poly-1** had a greater SEC-estimated molecular weight and broader molecular weight distribution. The aggregation phenomenon of both polymers is much more prominent than a reported poly(phenylenebutadiynylene) bearing more acidic urea functionalities (*D*_h_ = 25.3 nm in THF containing 0.1 vol% of DMSO) [28]. It is difficult to compare the aggregation behaviors of the two systems because the concentration of urea group in this study was several times higher than the reported one (13 μM), and the used solvent was completely different. It was likely that the concentration should be reduced to avoid the serious inter-polymer aggregation. Given that proper fluorescence intensity for anion sensing was needed, the polymer concentration was fixed at 22 mg L^−1^ for both polymers in DMF/H_2_O (v/v = 1:1) for all the following anion sensing determinations.

Next, the photoluminescence properties of the two polymers in the absence of anion addition were determined in comparison with that of monomer B (Figure 2(b)). The fluorescent emission of the polymers was observed at an excitation wavelength of 350 nm, as the maximum UV absorption was observed around this wavelength for both polymers. It was clear that both **Poly-1** and **Poly-2** showed an enhanced and red-shifted fluorescent emission than that of monomer B due to their extended conjugation length, though both of them were in a seriously self-aggregated state. This has been quite different from the fluorescent emission behavior of the above referred 3,5-bis(trifluoromethyl)phenyl-bonded urea functionalized poly(phenylenebutadiynylene) that showed almost complete fluorescence quenching phenomenon in a slightly aggregated state [28]. It is difficult to make a persuasive explanation why the fluorescent emission had been so different between the polymers in this study and the reported one, after all the conjugated main chain, the substituent, and the distance between neighboring urea groups were totally different. **Poly-1** showed an emission peak at 468 nm, exhibiting more remarkable red shift than **Poly-2** at 442 nm, mainly because the highly electron-donating amino group could stabilize the conjugation function of the polymer main chain, which decreased the π-π* energy gap during the charge transfer transitions.

**Figure 3.**
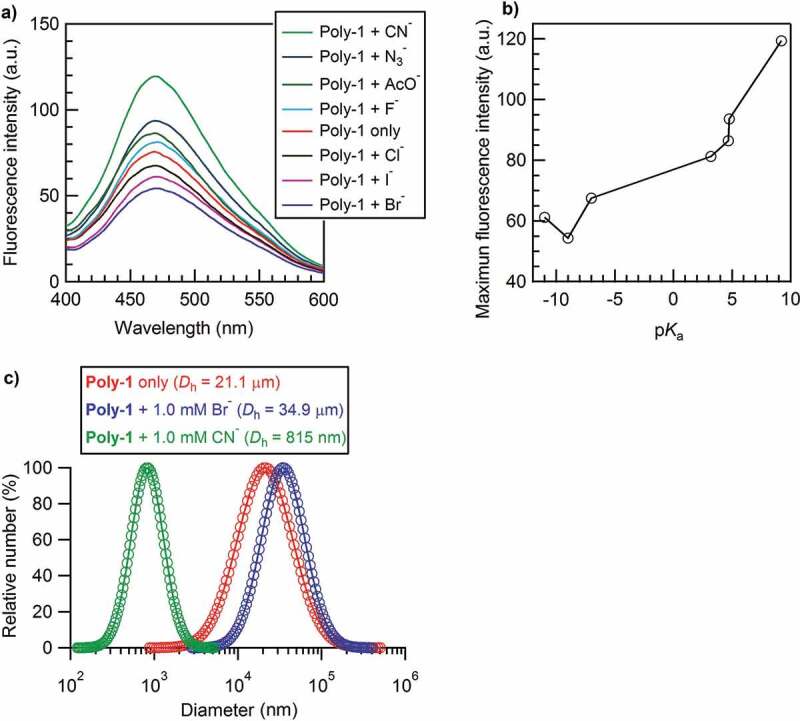
(a) Fluorescence spectra of **Poly-1** in the absence and presence of different anions under the conditions of **Poly-1** solution at 22 mg L^−1^ (the concentration of urea group was 60 μM) and anion solution at 1.0 mM in DMF/H_2_O (v/v = 1:1) at 25 °C and *λ*_ex_ = 350 nm. (b) Maximum fluorescent intensity at 468 nm of **Poly-1** + anion under the same conditions with (a). (c) Particle size distribution and average hydrodynamic diameter of **Poly-1** (red), **Poly-2** + Br^−^ (blue), and **Poly-2** + CN^−^ (green) determined by DLS in DMF/H_2_O (v/v = 1:1) under the same conditions with (a)

The photoluminescence properties of **Poly-1** (22 mg L^−1^, 60 μM urea group) in absence and presence of different anions, such as CN^−^, N_3_^−^, AcO^−^, F^−^, Cl^−^, I^−^, and Br^−^, were investigated at *λ*_ex_ = 350 nm under the anion solution at 1.0 mM in DMF/H_2_O (v/v = 1:1). The fluorescence spectra showed an emission peak at 468 nm for all samples, which indicated that the addition of an anion to **Poly-1** solution should have not brought about the change in the π-conjugation formation of main chain of **Poly-1**. It was observed that the peak photoluminescence intensity was clearly in a decreasing order as CN^−^ > N_3_^−^ > AcO^−^ > F^−^ > none > Cl^−^ > I^−^ > Br^−^, basically corresponding to the decreasing order of anion basicity, as shown in Table 1 and Figure 3(a,b). Given that the binding ability of the urea receptor is in general dominated by the basicity of anions [25,29], it is rational that a stronger binding ability between the basic anion and acidic urea group would result in disassembly of **Poly-1** aggregates and thus cause a greater fluorescent emission, namely **Poly-1** showing an aggregation-caused quenching (ACQ) effect. It was seen that the fluorescent changes in presence of anions had no regular relationship with the anion radius, both for the calculated and hydrated.

**Table 1.** Radius of anion and p*K*_a_ value of its conjugated acid

Anion (A)	Calculated radius *^a^* (Å)	Hydrated radius *^b^* (Å)	p*K*_a_ of conjugated HA	photoluminescence intensity at peak (a.u.)
CN^−^	1.91	3	9.21	119.40
N_3_^−^	1.95	–	4.7	93.65
AcO^−^	1.62	4–4.5	4.76	86.40
F^−^	1.19	3.5	3.2	81.25
Cl^−^	1.67	3	−7	67.50
Br^−^	1.82	3	−9	54.33
I^−^	2.06	3	−11	61.13

*^a^* (1) Jenkins, H.D.B.; Thakur, K.P.*J. Chem. Educ*.,**1979**, *56* (9), 576–577. (2) Website: http://www.wiredchemist.com/chemistry/data/thermochemical-radii-anions. *^b^* Kiclland, J. *J. Am. Chem. Soc*.**1937**, *59*(9), 1675–1678.

**Scheme 2.**
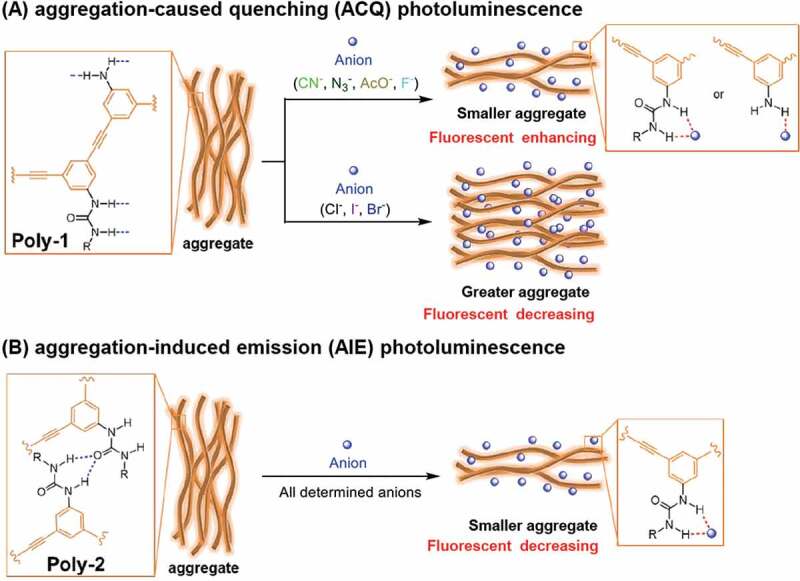
Schematic diagrams of (A) aggregation-caused quenching (ACQ) photoluminescence of **Poly-1** and (B) aggregation-induced emission (AIE) photoluminescence of **Poly-2.**

In order to confirm the above inference, DLS measurements of **Poly-1** in absence and presence of an anion were carried out to check how the anion influenced the aggregation behavior of **Poly-1**. As a consequence, **Poly-1** + CN^−^ with the maximum and **Poly-1** + Br^−^ with the minimum photoluminescence intensity were selected to do such investigations, as shown in Figure 3(c). Interestingly, the addition of the most basic CN^−^ to **Poly-1** caused a strong disassembly of **Poly-1** aggregates as the aggregate size of pure **Poly-1** (21.1 μm) decreased greatly to 815 nm (still aggregates). On the other hand, the addition of weakly basic Br^−^ to **Poly-1** caused a further assembly of **Poly-1** as the aggregate size of pure **Poly-1** (21.1 μm) increased to 34.9 μm. The addition of weakly basic Br^−^ caused a promoting effect on the assembly of **Poly-1**. Obviously, the disassembly of **Poly-1** aggregates brought about the enhancement of fluorescent emission and a further aggregation after weakly basic Br^−^ addition reduced photoluminescence intensity. It came to a conclusion that the π-conjugated **Poly-1** indeed displayed an aggregation-caused quenching (ACQ) photoluminescence effect, as the schematic diagram shown in Scheme 2(A). It should be noted that **Poly-1** formed very strong aggregation because a great excess amount of CN^−^ (1.0 mM, 16.7 times urea receptor) could not completely disassemble the H-bonds formed by the urea receptors of **Poly-1** (60 μM of urea group).

**Figure 4.**
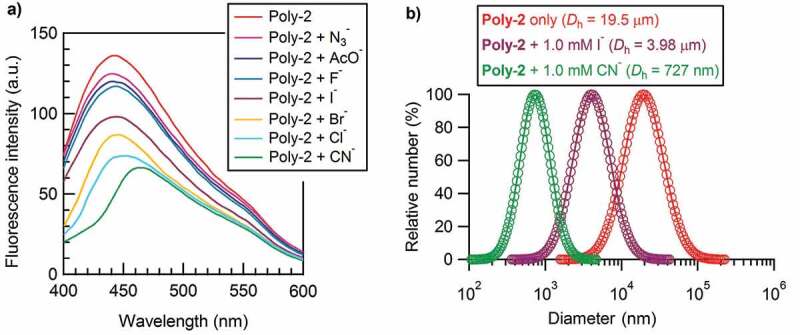
(a) Fluorescence spectra of **Poly-2** with different anions under the conditions of **Poly-2** solution at 22 mg L^−1^ (the concentration of urea group was 91 μM) and anion solution at 1.0 mM in DMF/H_2_O (v/v = 1:1) at 25 °C and *λ*_ex_ = 350 nm. (b) Particle size distribution and average hydrodynamic diameter of **Poly-2** (red), **Poly-2** + I^−^ (brown), and **Poly-2** + CN^−^ (green) determined by DLS in DMF/H_2_O (v/v = 1:1) under the same conditions with (a)

Similarly, the photoluminescence behavior of **Poly-2** in absence and presence of different anions, such as CN^−^, N_3_^−^, AcO^−^, F^−^, Br^−^, Cl^−^, and I^−^ were also investigated accordingly under the same conditions with **Poly-1** (the concentration of urea group was 91 μM for **Poly-2**). At this time, the addition of an anion to **Poly-2** decreased the photoluminescence for all anions, no matter how their basicity changed (Figure 4(a)). Among the seven detected anions, the addition of N_3_^−^, AcO^−^, F^−^, Br^−^, Cl^−^, or I^−^ only decreased the photoluminescence intensity but did not show any red or blue shift in photoluminescence peak. In sharp contrast, the addition of CN^−^ not only decreased the intensity most but also displayed a strong red shift from 442 nm to 464 nm at 1.0 mM CN^−^ (much clearer in Figure S6), which made its photoluminescence behavior significantly unique and selective (double-detection function) for CN^−^ sensing. The photoluminescence behavior of **Poly-2** in the presence of an anion varied greatly from that of **Poly-1**, and had been much different from the sensitive detection for fluoride or carboxylic anion in literatures [30–33]. It should be mentioned here that the addition of an anion to polymer solution did not bring about obvious color change for both polymer systems which showed slight yellow. Afterwards, the DLS measurements of **Poly-2** in the absence and presence of an anion in Figure 4(b) (CN^−^ and I^−^) and Figure S7 (N_3_^−^, AcO^−^, F^−^, Br^−^, and Cl^−^) indicated that the addition of anion could effectively disassemble the **Poly-2** aggregates, as the aggregation size of **Poly-2** (19.5 μm) after anion addition decreased to 11.3 μm for N_3_^−^, 8.23 μm for AcO^−^, 7.56 μm for F^−^, 3.98 μm for I^−^, 2.39 μm for Br^−^, 1.01 μm for Cl^−^, and 727 nm for CN^−^. Except CN^−^, it was found that the stronger basicity the anion, the greater average hydrodynamic diameter would be. This phenomenon has been opponent with our normal understanding and the reason is still not clear. To be interested, **Poly-2** obviously exhibited an aggregation-induced emission (AIE) behavior as proven above, which turned out to be opposite with **Poly-1** showing an ACQ behavior, as the schematic diagram shown in Scheme 2(B). The reason for the red shift of **Poly-2** after CN^−^ addition was ascribed to that the disassembly of **Poly-2** aggregates by destroying most of H-bonds between intermolecular urea receptors would result in the recovery of the distorted conjugated main chain, which enhanced the effective conjugation length and therefore decreased the π-π* energy gap during the charge transfer transitions.

**Figure 5.**
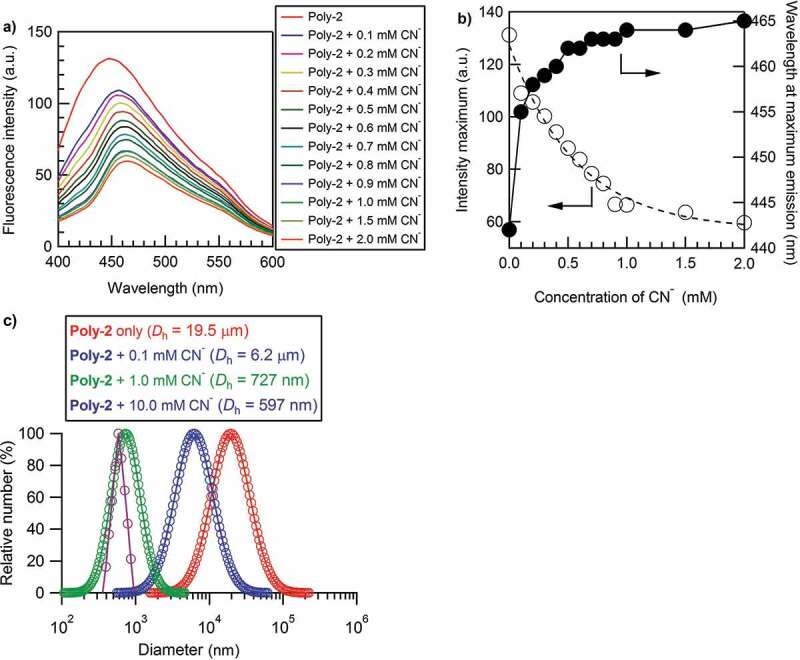
(a) Fluorescence spectra of **Poly-2** with cyanide anion at different concentrations under the conditions of **Poly-2** solution at 22 mg L^−1^ (the concentration of urea group was 91 μM) in DMF/H_2_O (v/v = 1:1) at *λ*_ex_ = 350 nm. (b) The dependence of fluorescence intensity maximum (left) and the wavelength at maximum emission (right) of **Poly-2** on the molar concentration of cyanide anion. (c) Particle size distribution and average hydrodynamic diameter of **Poly-2** (red), **Poly-2** + 0.10 mM CN^−^ (blue), **Poly-2** + 1.0 mM CN^−^ (green), and **Poly-2** + 10.0 mM CN^−^ (brown) determined by DLS in DMF/H_2_O (v/v = 1:1) under the same conditions with (a)

Given that **Poly-2** showed the AIE behavior and the selective photoluminescence sensing property for CN^−^, we focused our attention on CN^−^ and studied the photoluminescence titration experiments for the **Poly-2** + CN^−^ system to further examine the AIE effect and determine the detection sensitivity. The fluorescence spectra of **Poly-2** + CN^−^ by varying the anion concentration (Figure 5(a)) suggested that both of the photoluminescence intensity and emission peak continuously changed with increasing CN^−^ concentration. Specifically, the photoluminescence intensity decreased continuously and the emission peak red-shifted lastingly with the increase of CN^−^ concentration. The changes in maximum photoluminescence intensity and position (wavelength at emission peak) along with CN^−^ concentration are summarized in Figure 5(b). The intensity changes along with CN^−^ concentration could be well fitted using an exponential equation as follows.

$$I\;=\;56.84\;\left(a.u.\right)\;+\;70.57\;\left(a.u.\right)e^{-1.752c}$$

where *I* is the fluorescence intensity and *c* is the CN^−^ concentration in mM. The fitting curve could be severed as the detection line for CN^−^ when using photoluminescence intensity as the detection norm. On the other hand, the wavelength change at the emission peak was not as sensitive as photoluminescence intensity, especially at high concentration. Nevertheless, the wavelength change could be used as a supplementary detection parameter for CN^−^ sensing. The dependence of photoluminescence intensity on CN^−^ concentration implied that this system had high sensitivity in low concentration and became less sensitive when concentration was greater than 2.0 mM. The high sensitivity in low concentration for sensing the toxic CN^−^ is significantly important from a practical viewpoint. As for the lower detection limit, the spectral change in Figure 5(a) suggested that the lower detection limit was less than 0.1 mM. The very recent experiments gave a spectrally distinguishable detection limit of 0.01 mM (namely 10 μM) though the spectral curves varied in comparison with those in Figure 5(a) due to the further severe aggregation of **Poly-2** after a long preservation even in solid state, as shown in Figure S8. The detection limit of 10 μM is greater than five times that of colorimetric anion sensors based on urea-bearing π-conjugated polymer [30–33]. The improvement of the detection sensitivity is mainly attributed to the adopted photoluminescence method which is much more sensitive than colorimetric one.

To elucidate that the fluorescent quenching was caused by the AIE effect, the DLS measurements in Figure 5(c) were further carried out to elaborate the relationship between the photoluminescence intensity and the aggregated particle size of **Poly-2** by changing the CN^−^ concentration. With increasing CN^−^ concentration from 0 to 0.10, 1.0, and then 10.0 mM, it was clearly observed that the photoluminescence intensity decreased as afore discussed, while the aggregated particle size showed an exponential decline from 19.5 μm, to 6.2 μm, to 727 nm, and at last to 597 nm. Obviously, the more disassembled, the weaker photoluminescence intensity **Poly-2** displayed, indicating once more that **Poly-2** had an AIE effect.

**Figure 6.**
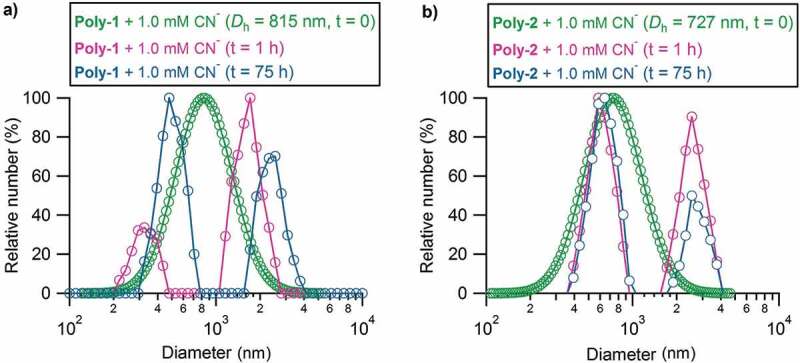
The time dependence of particle size distribution and average hydrodynamic diameter of **Poly-1** and **Poly-2** at 22 mg L^−1^ (the concentration of urea group was 60 μM for **Poly-1** and 91 μM for **Poly-2**) with 1.0 mM CN^−^ estimated by DLS in DMF/H_2_O (v/v = 1:1) at t = 0 h (green), t = 1 h (light red), and t = 75 h (light blue)

The aggregation behavior of both polymers was found to be temporal and very complicated in both solid and solution states. The study on solid state is omitted and we only check the time-related aggregation states by DLS measurements. The time dependence of particle size distribution and average hydrodynamic diameter of **Poly-1** and **Poly-2** (22 mg L^−1^) with 1.0 mM CN^−^ at t = 0 h, 1 h, and 75 h were recorded in Figure 6. It was observed that the freshly prepared polymer solution formed monodistributed aggregates for both polymers, but then dynamically transformed to the double-distributed aggregates by simultaneously forming a smaller and a larger aggregate after 1 h. After 75 h, the sizes of both aggregates became larger for **Poly-1**, while kept unchanged for **Poly-2**. Moreover, the containment of the smaller aggregate increased for both polymers in presence of a large excess amount of CN^−^. The polyphenyleneethynylene-induced aggregation behavior showed great dynamic nature, which made the discussion on this point difficult. This has also been the reason why the determination of photoluminescence property was changeable after sample storage even in solid state. The complete disassembly of the polymer aggregates could not be achieved at even an extremely high CN^−^ concentration of 10.0 mM after three days, indicating the lasting formation of time-related dynamic intermolecular H-bonds for both polymers. For this reason, an intensive investigation on the dynamic time-dependent photoluminescence property and aggregation state of **Poly-2** + 1.0 mM CN^−1^ in DMF/H2O (v/v = 1:1) was implemented to confirm the relatively stable equilibrium state to be reached, as shown in Figure S9. It was found that both the fluorescent intensity and particle size varied dramatically within the initial 3 h and the whole system tended to an almost unchanged equilibrium state after 3 h. This result suggests that our determinations after several-hour (at least 3 h) deposition of polymer solution were reasonable and acceptable for fluorescence measurements.

## Conclusions

The π-conjugated polyphenyleneethynylene, **Poly-2**, bearing urea receptor in this study showed photoluminescence responsivity for all studied anions. In particular, it showed special selectivity and sensitivity for probing CN^−^. Its selectivity has been reflected on the double-detection function as the addition of CN^−^ to **Poly-2** solution not only decreased the intensity most but also displayed a strong red shift. The probing lower limitation could be low as 10 μM. This detection limitation has been a moderate value among the reported ones as some small molecular probes have been disclosed to detect much lower concentrations [17,18,23], but this detection limitation is sufficient enough for human beings as the blood cyanide concentrations reach a poisoning level at 23–26 μM. To be interested, **Poly-2** showed AIE-type photoluminescence property, which has been rarely revealed for the π-conjugated polyphenyleneethynylene polymers. Its precursor polymer, **Poly-1**, on the contrary showed an ACQ-type photoluminescence property. DLS experiments indicated that both **Poly-1** and **Poly-2** underwent serious inter-polymer self-aggregation even in high dilution or under anion addition conditions, and the assembly and disassembly of the polymer chains were dynamically changing with time. The results in this study are believed to be capable of enriching the concept of urea-bearing polymer probes.

## Supplementary Material

Supplemental Material
