# Supplementary material for: Aggregation-induced fluorescent response of urea-bearing polyphenyleneethynylenes toward anion sensing
Source: Sci Technol Adv Mater. 2021 Jun 16;22(1):597–606. doi: 10.1080/14686996.2021.1942982 (PMC8344232; doi:10.1080/14686996.2021.1942982)
Supplement: Supplemental Material [file TSTA_A_1942982_SM6663.pdf]

# Aggregation-induced Fluorescent Response of Urea-bearing Polyphenyleneethynylenes toward Anion Sensing

Jian Li,[a,b] Muhammad Saleem,[a] Qian Duan,[c] Toyoji Kakuchi,[c,d] and Yougen Chen[a*]

[a] Institute for Advanced Study, Shenzhen University, Shenzhen, 518060, China

[b] College of Physics and Optoelectronic Engineering, Shenzhen University, Shenzhen, 518060, China

[c] School of Materials Science and Engineering, Changchun University of Science and Technology, Weixing Road 7989, Jilin, 130022, China

[d] Frontier Chemistry Center, Faculty of Engineering, Hokkaido University, Sapporo, Hokkaido, 060-8628, Japan

CORRESPONDING AUTHOR FOOTNOTE

Tel & Fax: +86-75526943283

E-mail: chenyg@szu.edu.cn

# Experimental Section

**Materials.** Dichloromethane ($CH_2Cl_2$, >99%), acetone (>98%), deuterated chloroform ($CDCl_3$,>99.8%), acetone-$d_6$ ($CD_3COCD_3$,>99.8%), *n*-hexane (98%), tetrahydrofuran (THF,>99%), methanol (MeOH, >98%), toluene (99%), triethylamine (>99.0%), 3,5-dibromoaniline, triisopropylsilylacetylene, trimethylsilylacetylene andsodium hydride (60 wt% in mineral oil) were purchased from TCI Chemicals. Hexyl isocyanate, bis(triphenylphosphine)palladium(II) dichloride were purchased from Aldrich. All other reagents were used as received. Toluene and THF were distilled over Na/benzophenone under an argon atmosphere and degassed by three freeze-pump-thaw cycles prior to use. All other chemicals were purchased from available suppliers and used without further purification.

**Measurements.** The polymerization was carried out in an MIKROUNA stainless steel glove-box equipped with a gas purification system under a dry argon atmosphere ($H_2O$, $O_2$<0.01 ppm). The moisture and oxygen contents in the glove box were monitored by an MB-MO-SE 1 and an MB-OX-SE 1 sensor, respectively. The $^1$H and $^{13}$C NMR spectroscopy (600 MHz) was recorded using a BRUKER ASCEND TM 600, BrukerBiospin GmbH Rheinstetten, Germany. Size exclusion chromatography (SEC) in DMF was performed at 40 °C at the flow rate of 0.35 mL min$^{-1}$ using a TosohHLC-8320 GPC System equipped with two TSK gel Super Multipore HZ-M columns (4.6 mm I.D. × 15 cm × 2), an EcoSEC GPC System with an RI detector(+,0.5s), a UV-8320 detector(254nm,+0.5s), a GPC Workstation EcoSEC-WS, and an Auto sampler, to which 10μL of sample is injected with the concentration of 0.2wt%.The number-average molecular weight ($M_{n,SEC}$) and polydispersity ($M_w/M_n$) of the polymers were calculated based on polystyrene standards. The UV-vis absorption spectra of the polymers were measured by a UV-Vis spectrophotometer (Routine UV-Vis, Cary 60, Agilent) and the fluorescence spectra were

recorded using a fluorescence spectrophotometer (RF-5301PC, Shimadzu). The particle size distribution and average hydrodynamic diameter were measured by a dynamic light scattering method (NanoBrook 90PlusPALS zeta sizer, Brookhaven Instruments). FT-IR spectra were recorded on a Perkin Elmer Frontier. Samples fashioned into KBr pellet disks were placed into a holder for transmission IR spectral analysis. Spectra of mid infrared from an accumulation of 16 scans in the range of 4000–450 $cm^{-1}$ were recorded.

**Synthesis of 3,5-bis(triisopropylsilylethynyl)aniline**

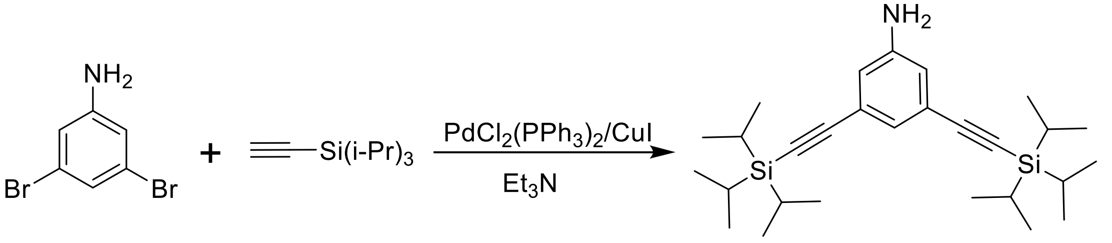


15.00 g (59.71 mmol) 3,5-dibromoaniline, 3.96 (5.64 mmol) $Pd(PPh_3)_2Cl_2$ and 2.16 g (11.32 mmol) CuI were dissolved in a mixture of 140 mL triethylamine and 150 mL toluene. The mixture was heated to 80 °C under argon. 21.96 g (117.78 mmol) triisopropylsilyl) acetylene was added dropwise (orange suspension turns dark brown) and the mixture was stirred at 80 °C over night. Water was added to the mixture and the organic phase was washed with satd. $NH_4Cl$ (aq), 1 N HCl(aq), 10% $Na_2CO_3$ (aq), and dried over $MgSO_4$. After evaporation of the solvent, the crude product was purified by column chromatography using firstly hexane and then a 10:1 mixture of petrol ether and ethyl acetate as eluent to yield 22.71 g (86%) of the pure compound as viscous yellow oil. $^1$H NMR (500 MHz, $CDCl_3$) d 7.0 (s, 1H, aromatic H), 6.73 (s, 2H, aromatic H), 3.65 (s, 2H, amino H), 1.18 (s, 42H, H from $^i$Pr). $^{13}$C NMR (125 MHz, $CDCl_3$)

146.95 (aromatic C, 1C), 126.5 (aromatic C, 1C), 124.76 (aromatic C, 2C), 118.0 (aromatic C, 2C), 107 (acetylene C, 2C), 90 (acetylene C, 2C), 19.30 ($^{i}$Pr C, 6C), 12.45 ($^{i}$Pr C, 12C).

**Deprotection of 3,5-bis((triisopropylsilyl)ethynyl)aniline**

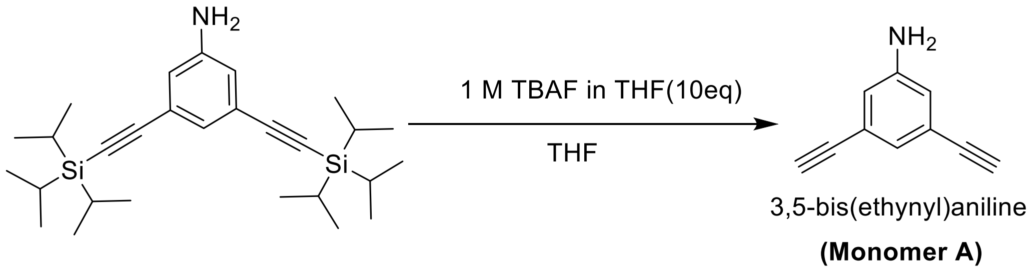


Deprotection of 3,5-bis(triisopropylsilylethynyl)aniline was achieved by desilylation using tetra-*n*-butylammonium fluoride (TBAF) in THF promoted by a small amount of $CH_3OH$. $^{1}$H NMR (500 MHz, $CDCl_3$) 6.95 (s, 1H, aromatic H), 6.65 (s, 2H, aromatic H), 3.65 (s, 2H, amino H), 2.95 (s, 2H, acetylene H).

**Synthesis of 1-(3, 5-dibromophenyl)-3-hexylurea**

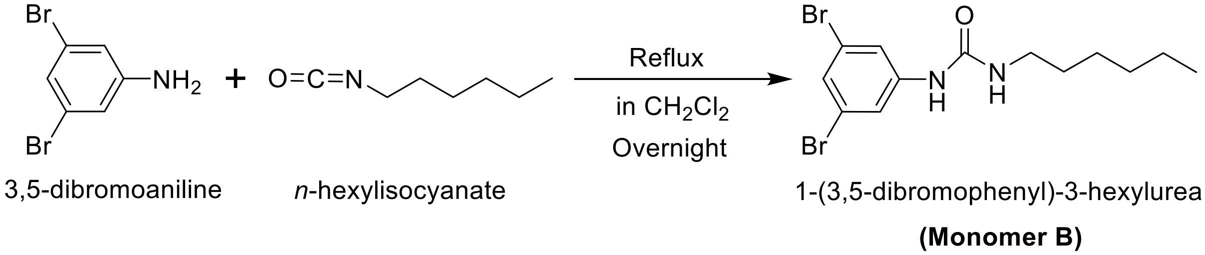


Hexyl isocyanate (3.08 g, 24.2 mmol) was added dropwise to the stirring solution of 3, 5-dibromoaniline, 4.12 (20.72 mmol) in 60 mL of dichloromethane at low temperature using ice bath. After complete addition the reaction mixture was then allowed to stir at room temperature for an hour and then reflux for 6 hours, during which time a precipitate appeared. The solvent was then removed by rotavapor and the resulting urea compound was purified by recrystallization from ethanol at low temperature to obtain urea derivative as a white crystalline

solid.$^1$H NMR (500 MHz, $CDCl_3$) δ 8.15 (s, 1H, urea H), 7.65 (s, 2H, aromatic H), 7.15 (s, 1H, aromatic H), 5.75 (s, 1H, urea H), 3.10 (m, 2H, H from hexyl), 1.4 (m, 2H, H from hexyl), 1.25 (s, 6H, H from hexyl), 0.75 (s, 3H, H from hexyl).

**Polymerization procedure of Poly-1 and Poly-2**

All polymerizations were carried out in a glass tube equipped with a three-way stopcock under oxygen. DMF (0.4 mL) and $Et_3N$ (0.6 mL) were added to a mixture of monomers (0.05 mmol), $Pd(PPh_3)_2Cl_2$ (3.5 mg, 0.005 mmol), $PPh_3$ (1.3 mg, 0.005 mmol) and CuI (0.95 mg, 0.005 mmol) under argon, and the resulting solution was kept at 80 °C for 20 h. Then, the reaction mixture was poured into a large volume of diethyl ether to precipitate the polymer. It was separated by filtration using a membrane filter and dried under reduced pressure. $^1$H NMR (500 MHz, $CDCl_3$): 6.10-8.65 (m, amide and ring protons), 0.5-1.5 (m, $CH_3$ and $CH_2$ protons), 7.60-7.74 (s, 2H, Ar). $M_{n,SEC}$ = 13,500 g mol-1, $M_w/M_n$ = 2.42 for **Poly-1**. Yield >90%. **Poly-2** was sequentially obtained by directly reacting Poly-1 with hexyl isocyanate followed by precipitation in methanol. $M_{n,SEC}$ = 6,090 g mol-1, $M_w/M_n$ = 1.43, almost quantitative yield.

**Sample preparation for UV-vis and fluorescent measurements.** 4.4 mg polymer (**Poly-1** or **Poly-2**) was first dissolved in 50 mL DMF and then diluted to 100 mL with distilled water to meet the desired stock concentration of 44 mg $L^{-1}$. The prepared stock solution of anion in DMF/$H_2O$ = 1:1 (v/v) was directly mixed with the polymer solution at 1:1 (v/v) with shaking to ensure the polymer concentration of 22 mg $L^{-1}$ and the anion concentration was adjusted accordingly by dilution from concentrated solution. For the UV-vis and fluorescent analyses, the solution was deposited for several hours (at least 3 h), after which measurements were carried out.

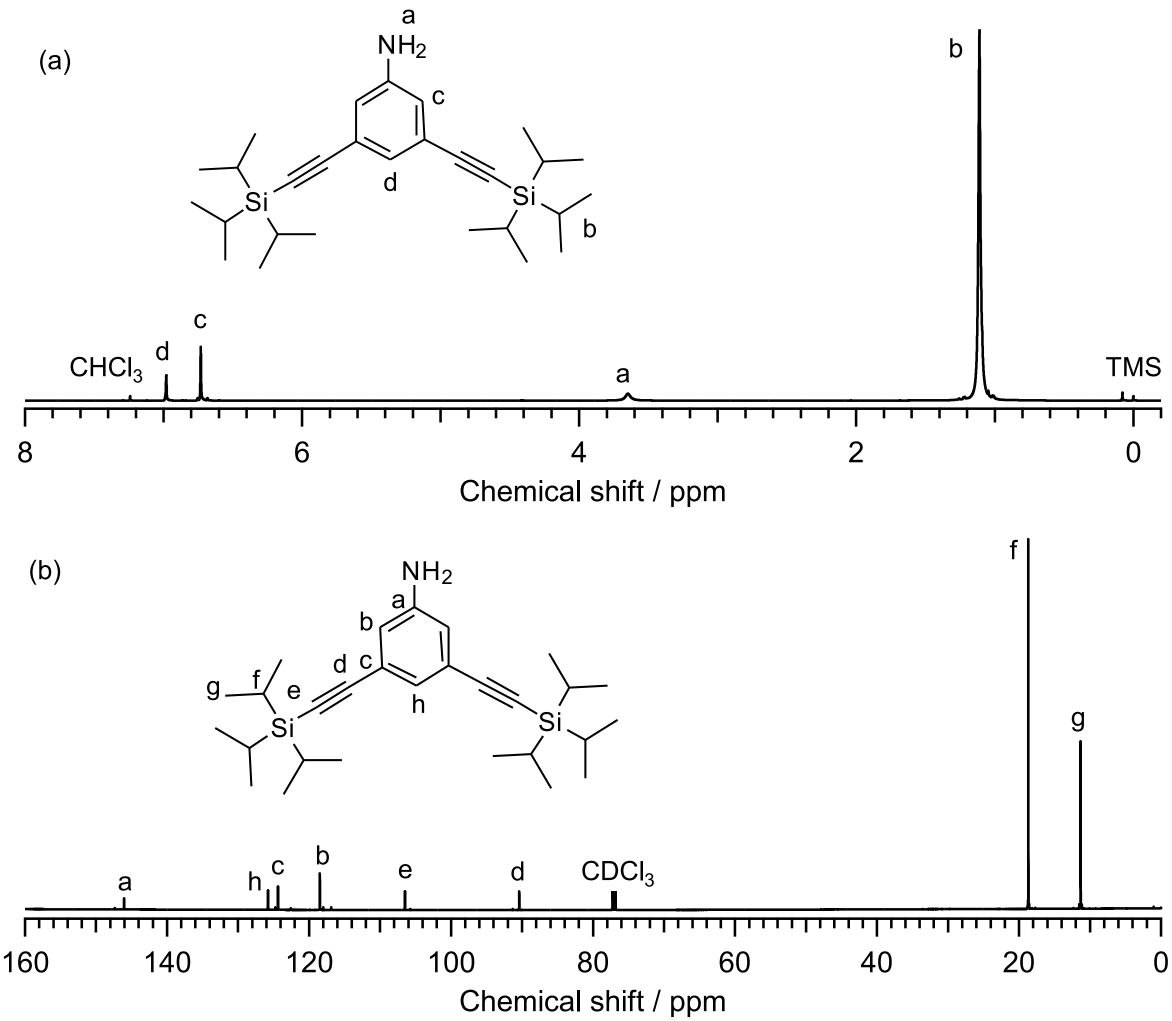


**Figure S1.** $^{1}H$ (a) and $^{13}C$ (b) NMR spectra of 3,5-bis(triisopropylsilylethynyl)aniline in $CDCl_3$.

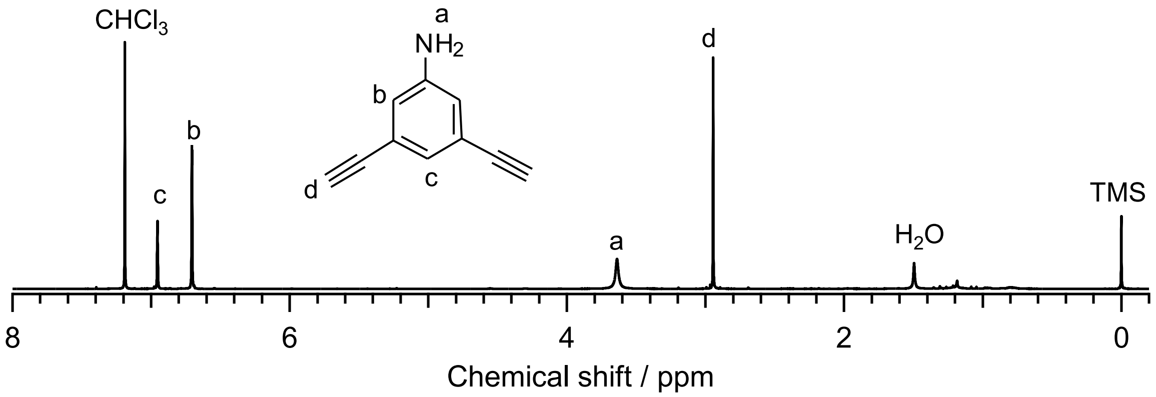


**Figure S2.** $^1$H-NMR spectrum of 3,5-diethynylaniline in $CDCl_3$.

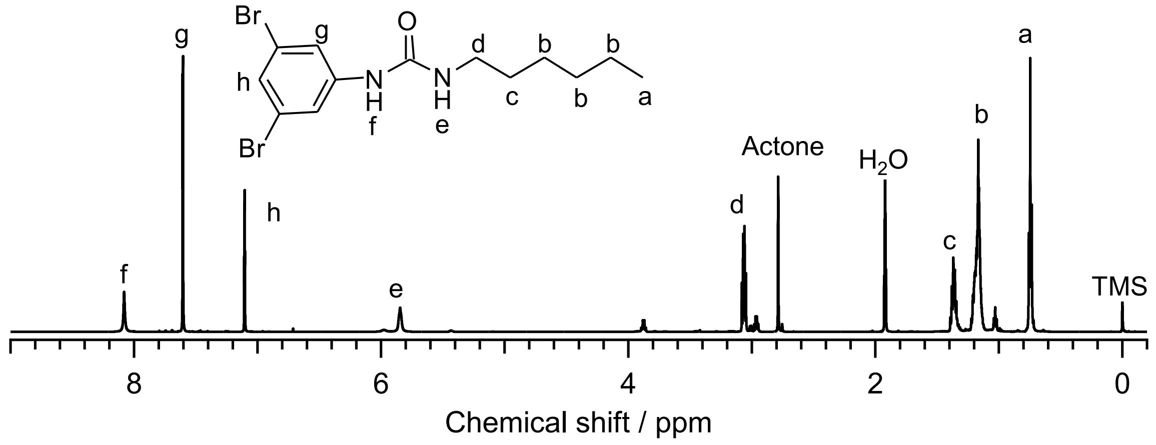


**Figure S3.** $^1$HNMR spectrum of 1-(3, 5-dibromophenyl)-3-hexylurea in actone-$d_6$.

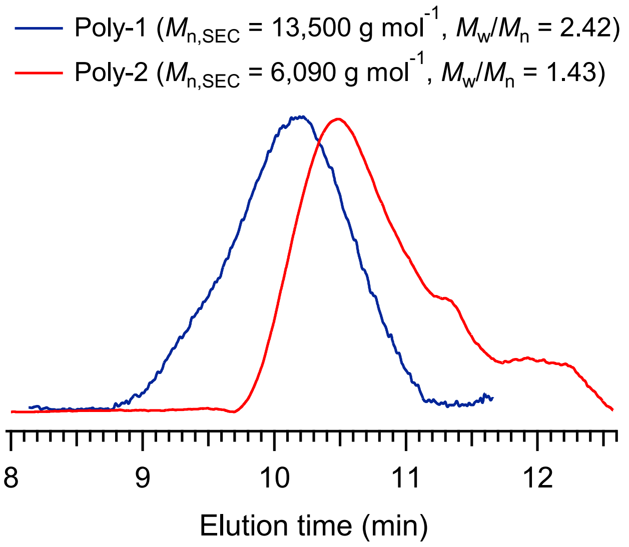


**Figure S4.** SEC traces of **Poly-1** and **Poly-2** (eluent, 0.01 mol L$^{-1}$ LiBr in DMF; flow rate, 0.35 mL min$^{-1}$).

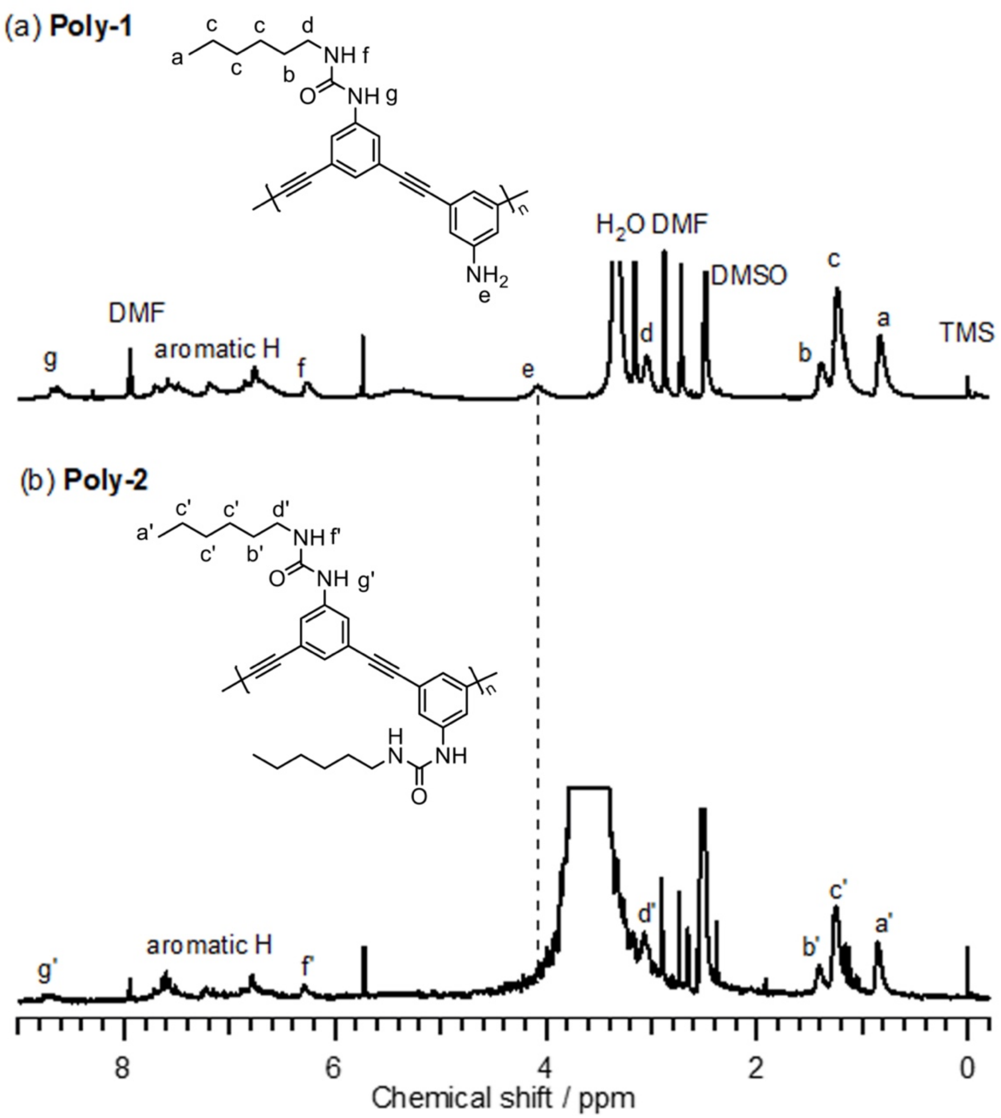


**Figure S5.** $^1$H-NMR spectra of **Poly-1** and **Poly-2** determined in DMSO-$d_6$.

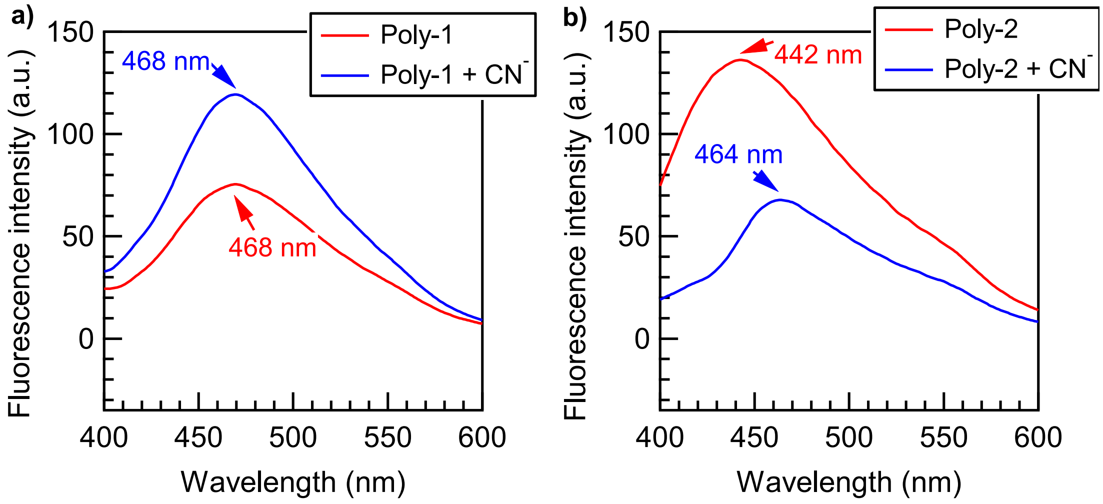


**Figure S6.** Fluorescence spectra of **Poly-1** and **Poly-2** before (red) and after (blue) addition of $CN^{-}$ under the condition of 0.01 mM polymer solution and 1.0 mM anion solution in DMF/$H_2O$ (v/v = 1:1).

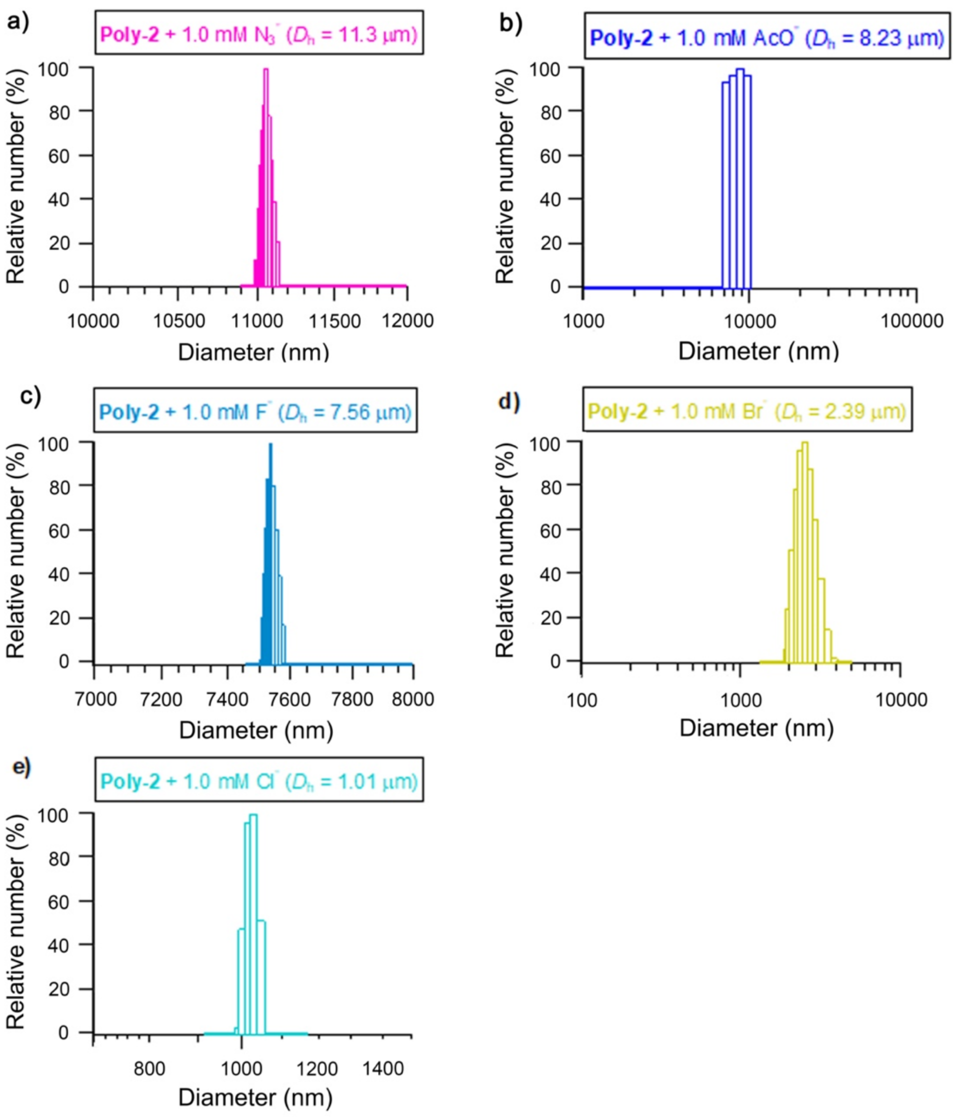


**Figure S7.** Particle size distribution and average hydrodynamic diameter ($D_h$) of **Poly-2** + anion determined by DLS in DMF/$H_2O$ (v/v = 1:1) mixed solvent at 25 °C under the weight concentration of 22 mg $L^{-1}$ for **Poly-2** (the concentration of urea group was 91 μM for **Poly-2**) and the anion concentration of 1.0 mM: (a) **Poly-2** + $I^-$, (b) **Poly-2** + $AcO^-$, (c) **Poly-2** + $F^-$, (d) **Poly-2** + $Br^-$, and (e) **Poly-2** + $Cl^-$.

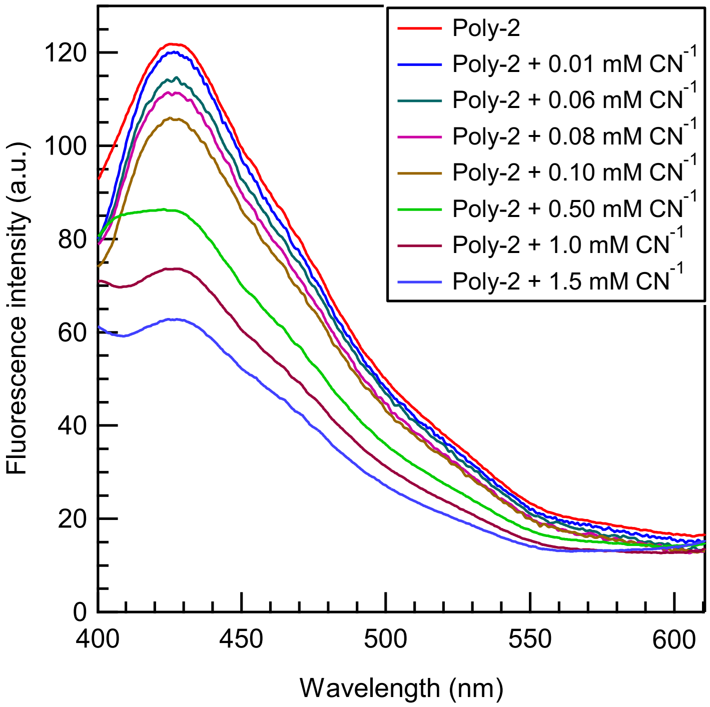


**Figure S8.** Fluorescence spectra of **Poly-2** with cyanide anion at different concentrations under the conditions of **Poly-2** solution at 22 mg $L^{-1}$ (the concentration of urea group was 91 μM) in DMF/$H_2O$ (v/v = 1:1) at $\lambda_{ex}$ = 350 nm.

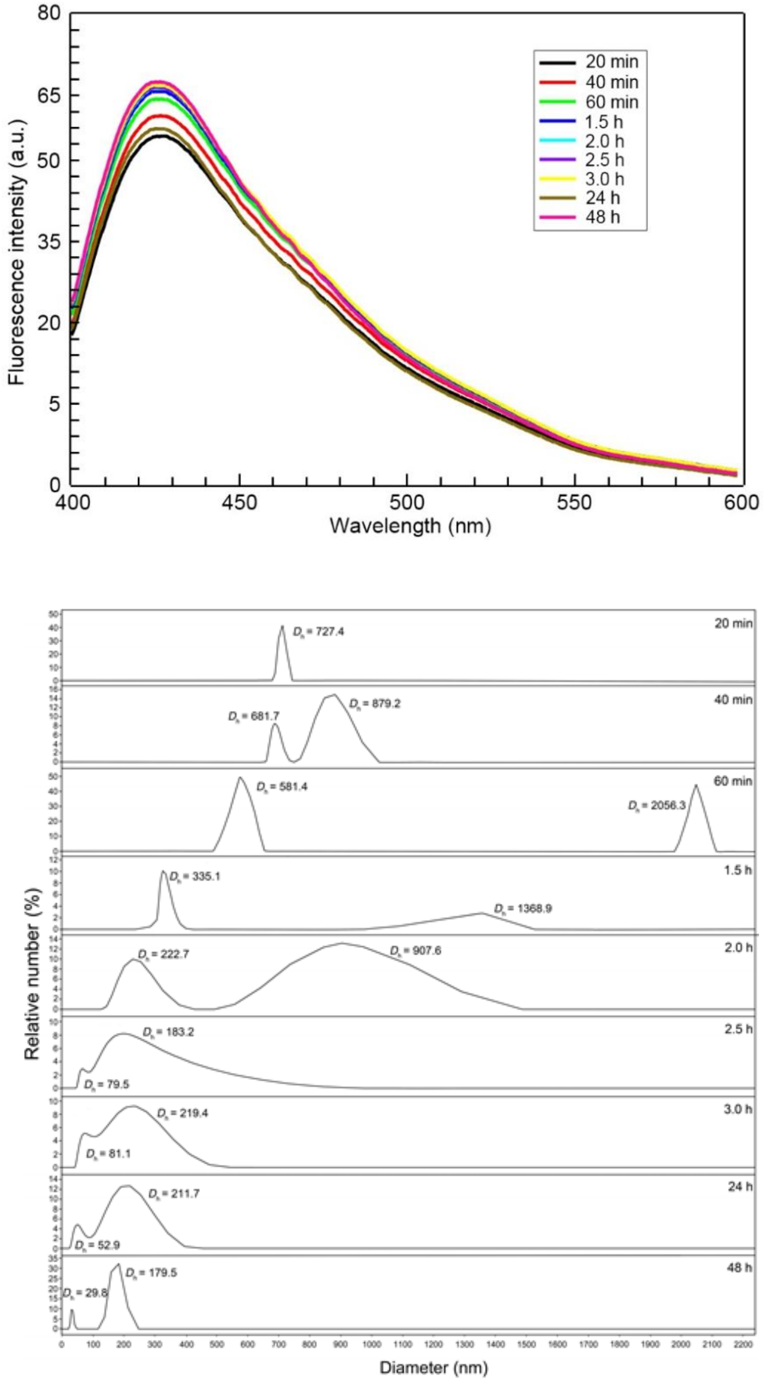


**Figure S9.** Time-dependent fluorescence spectra (above) and particle size distributions (below) of **Poly-2** with cyanide anion under the conditions of **Poly-2** solution at 22 mg $L^{-1}$ (the concentration of urea group was 91 μM) in DMF/$H_2O$ (v/v = 1:1) at $\lambda_{ex}$ = 350 nm.
